# Coronary artery aneurysms in children is not always Kawasaki disease: a case report on Takayasu arteritis

**DOI:** 10.1186/s41927-021-00197-0

**Published:** 2021-08-12

**Authors:** Michelle Lee, Esra Meidan, MaryBeth Son, Audrey Dionne, Jane W. Newburger, Kevin G. Friedman

**Affiliations:** 1 Department of Pediatrics, Harvard Medical School, Boston, MA USA; 2 Department of Cardiology, Boston Children’s Hospital, 300 Longwood Avenue- Farley 2, Boston, MA 02115 USA; 3 Division of Immunology, Boston Children’s Hospital, Boston, USA

**Keywords:** Kawasaki disease, Takayasu’s arteritis, Coronary artery aneurysm, Case report

## Abstract

**Background:**

Coronary artery (CA) aneurysms in children are a rare but potentially life-threatening finding and are highly associated with Kawasaki disease (KD).

**Case presentation:**

We describe a four-year-old female with a vasculitis and CA aneurysms. She had a prolonged course with recurrent fever and systemic inflammation several times upon discontinuation of steroid treatment. Due in part to the CA aneurysms, she initially was diagnosed with KD but due to the unusual clinical course, further evaluation was performed. Abdominal and chest MRI/A revealed diffuse aortitis suggestive of a large vessel vasculitis, specifically Takayasu arteritis. With treatment targeted for Takayasu arteritis, there was resolution of fever and inflammation and the CA aneurysms improved.

**Conclusions:**

This case demonstrates the utility in broadening the differential diagnosis in cases of presumed KD with CA involvement in which the clinical course is atypical for KD.

## Background

Kawasaki disease (KD), the leading cause of acquired pediatric heart disease in the developed world, is a vasculitis that preferentially affects medium-sized arteries, particularly the coronary arteries (CA) [1]. Coronary artery involvement can range from transient dilation to large/giant aneurysms. Using American Heart Association z-score criteria, ~ 10–30% of KD patients develop CA dilation or aneurysm despite appropriate IVIG treatment [2, 3]. Due to the strong association between KD and CA aneurysms, febrile and inflamed children who are found to have CA dilation or aneurysm are almost always diagnosed and treated for KD. This case illustrates that although KD is the most common etiology of CA aneurysm in children [1], it is not the only potential cause of fever, inflammation, and CA aneurysm in the pediatric population.

## Case presentation

A 4-year-old female patient presented to her pediatrician with 2 days of fever and emesis. She was initially treated with amoxicillin for presumptive strep throat. Fever continued despite antibiotic treatment and she represented to an outside hospital’s emergency department (Day 5 of illness). By this time, she had developed a rash, hand puffiness and bilateral non-exudative conjunctivitis. She was given a 3 day course of corticosteroids. She defervesced and her symptoms resolved.

The fever recurred 10 days after initial symptom onset and she was admitted. Labs were notable for an erythrocyte sedimentation rate (ESR) of 53 mm/hr., c-reactive protein (CRP) 3.9 mg/dL, white blood count (WBC) 23.4 × 10^3^ cells/uL, hemoglobin (Hgb) 11.9 g/dL, platelets 393 × 10^3^ cells/uL, sodium 132 mEq/L, and multiple negative SARS-CoV-2 antibody and PCR tests. Echocardiogram was performed due to concern for KD and was normal, including normal CA size and appearance. She was treated on Day 11 with 2 g/kg of intravenous immunoglobulin (IVIG) and high dose aspirin for presumed KD. Her fever resolved and she was discharged home on Day 13. Three days later she had recurrent low-grade fevers and elevated inflammatory markers which prompted readmission and retreatment with IVIG (Day 16). Her physical exam at that time was notable for bilateral conjunctival injection, normal tongue and oropharynx without erythema or lesions, no lymphadenopathy, pulses intact throughout with capillary refill less than 2 s, “reluctant but full” joint range of motion, mild edema of hands and feet, no fingertip or toe peeling, and no rash. Labs at this admission suggesting incomplete KD were hypoalbuminemia 2.7 mg/dL, elevated CRP to 14.4 mg/dL, elevated ESR 130 mm/hr., normocytic anemia with Hgb 10.8 g/dL, leukocytosis to17.4 Th/uL, and mild thrombocytosis to 486 Th/uL Her echocardiogram showed a new, small, saccular aneurysm in the left anterior descending (LAD) CA with maximal dimension of 2.9 mm, z-score 3.27 (Day 17). She met criteria for incomplete KD based on the American Heart Association incomplete KD algorithm with 3/5 clinical criteria, elevated inflammatory markers and abnormal coronary arteries. A second dose of IVIG (2 g/kg) was given but fever persisted and oral corticosteroids were reinitiated at 1 mg/kg BID. She subsequently defervesced and was discharged on oral steroids and low dose aspirin.

**Fig. 1:**
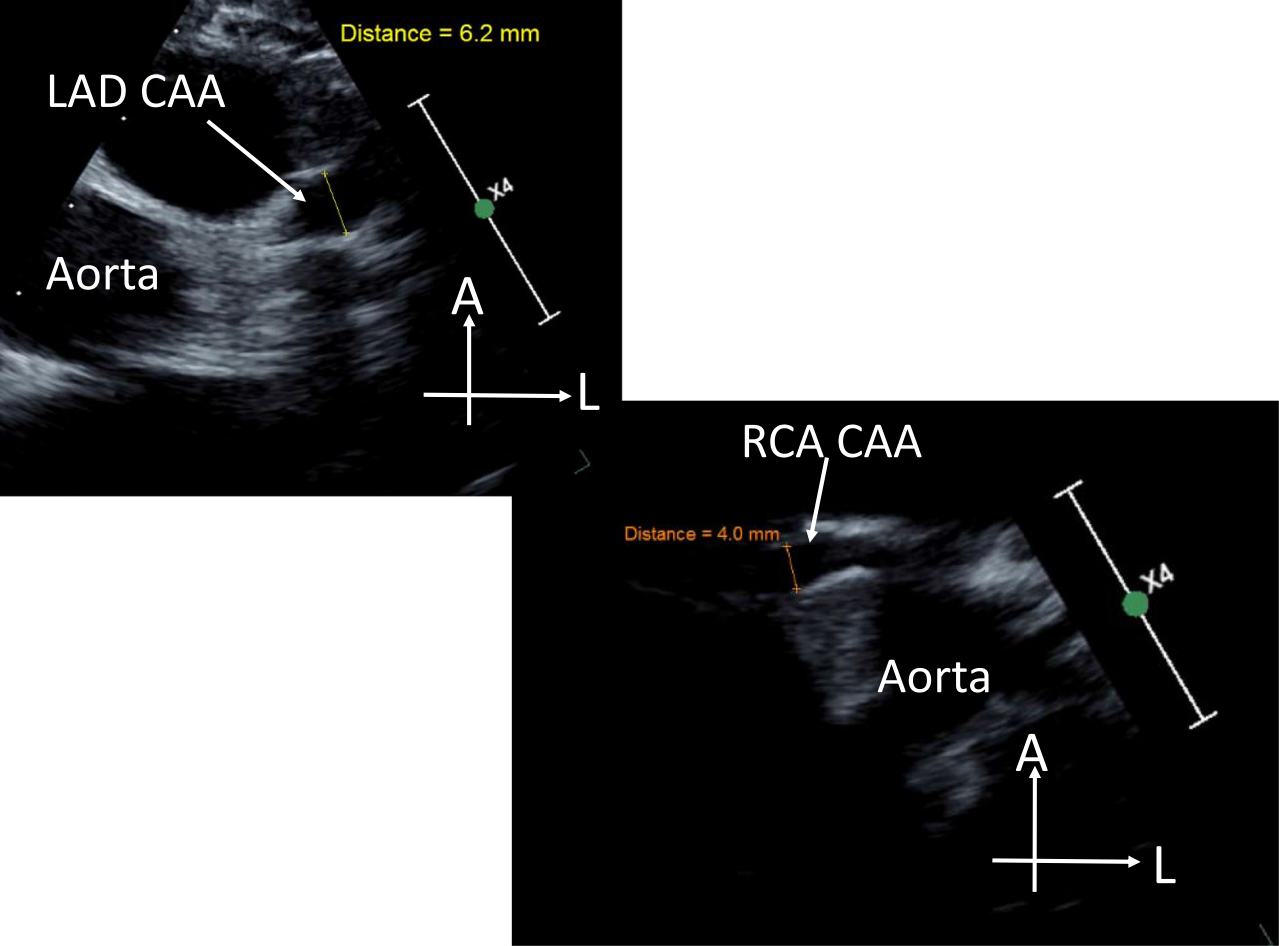
Echocardiogram images showing largest coronary artery aneurysms over follow-up. **a** Large/giant, saccular left anterior descending coronary artery aneurysm measuring ~ 6 mm. **b** Moderate-sized, fusiform proximal right coronary artery aneurysm. LAD, left anterior descending; CAA, coronary artery aneurysm; RCA, right coronary artery; A, anterior; L, left

Over serial outpatient follow-up, she had interval increases in CA measurements on echocardiography (Days 21, 24, and 29), with LAD dimension progressing to 4.5 mm, z-score 9.0 and RCA dimension to 3.5 mm, z-score 4.2. She was weaned off steroids over 3 weeks, but developed recurrent fever and increase in CRP (Day 37), prompting readmission. Over this course she was noted to have mild systemic hypertension. Echocardiogram and CT were notable for an interval increase of the previously noted LAD aneurysm, now measuring 5–6 mm (z-score ~ 11) as well as RCA aneurysm with max dimension ~ 4 mm (Fig. 1). On Day 38, she was restarted on corticosteroids and received cyclophosphamide 10 mg/kg IV for presumed diagnosis of refractory KD. CRP normalized, repeat echo was stable, and she was discharged with a plan for steroid wean (Day 39–54).

**Fig. 2:**
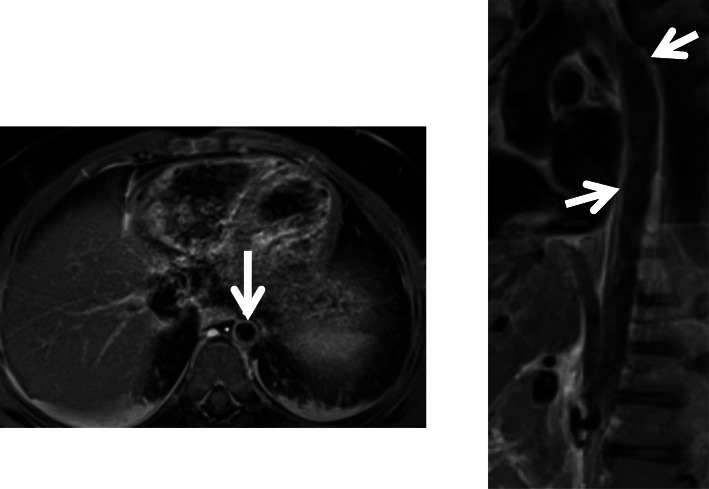
Magnetic Resonance Imaging Angiogram. **a** Axial view showing diffuse thickening and abnormal enhance of the thoracic and abdominal aortic walls indicative of diffuse aortitis (arrow) **b**, Sagittal long-axis view showing diffuse thickening (arrows) and abnormal enhance of the thoracic and abdominal aortic walls indicative of diffuse aortitis

**Fig. 3:**
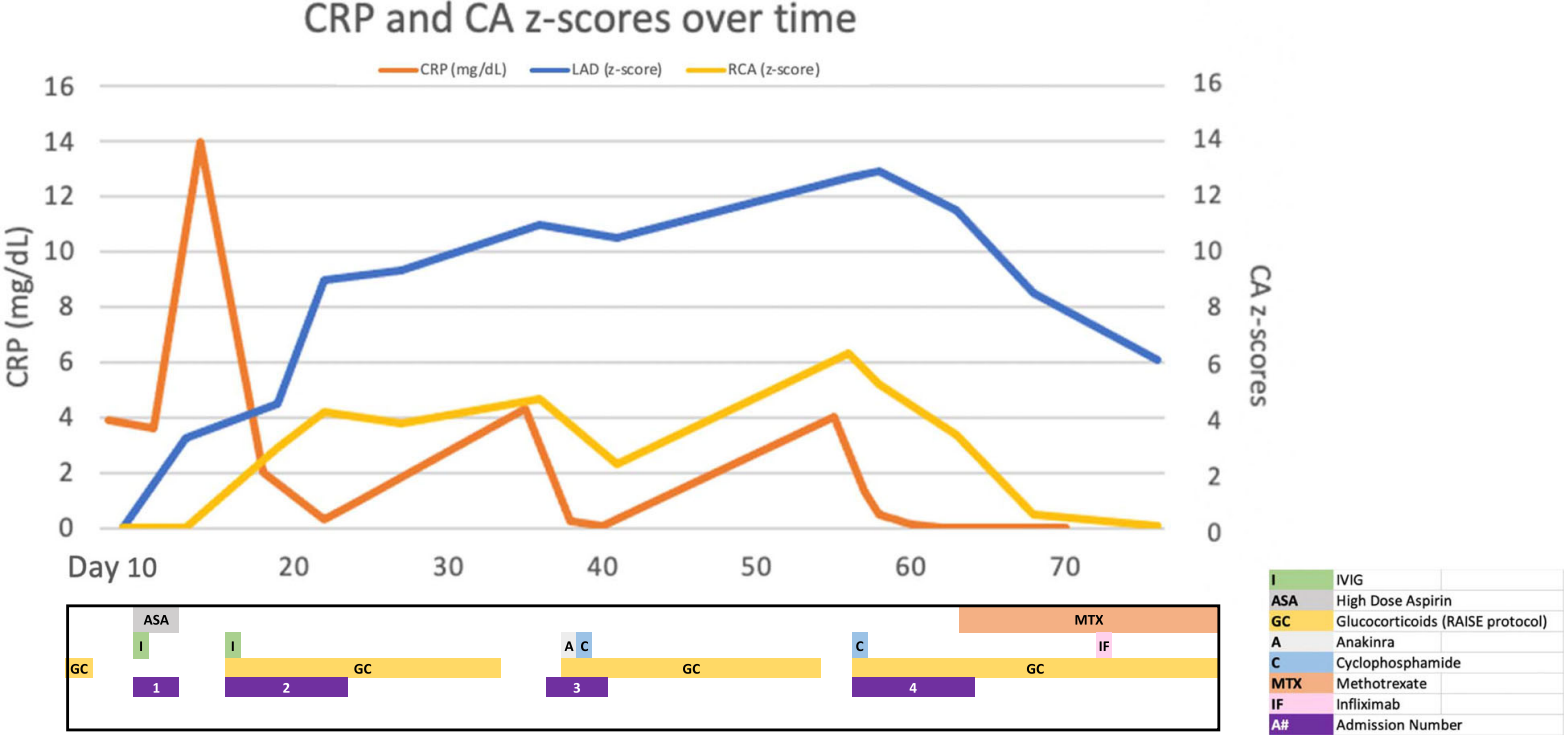
Coronary Artery z-scores, CRP, and Treatments over Follow-up. LAD, left anterior descending coronary artery, RCA right coronary artery

Three days after completion of the second steroid wean (Day 57) she developed recurrent fever and elevated inflammatory markers prompting repeat admission and treatment with a second dose of cyclophosphamide (10 mg/kg) and re-initiation of steroids. Given her prolonged and atypical course of presumed KD, with recurrence of inflammation and fever with steroid weans she was evaluated for alternative forms of vasculitis. This included an MRI/A abdomen that showed diffuse wall thickening and diffuse abnormal enhancement of the thoracic and abdominal aorta with suspected involvement of the right brachiocephalic and bilateral common carotid arteries, indicating a diffuse aortitis (Fig. 2). KD does not lead to diffuse aortitis (i.e., large vessel vasculitis) and based on the MRA as well as the clinical course so alternative diagnoses were considered. In this case, the most likely etiology of a chronic, large vessel vasculitis with a relapsing course upon steroid wean was felt to be Takayasu’s arteritis (TA). She met TA diagnostic criteria, using the Pediatric Rheumatology European Society criteria, based on angiographic findings and systemic hypertension. Given the diagnosis of TA, she was discharged on oral methotrexate and prednisolone with plan for infliximab infusions treatment. Over 2 months of follow-up on this regimen, she clinically improved with no recurrence of fever or laboratory evidence of inflammation. Her most recent echocardiogram showed a decrease in the size of the LAD aneurysm (3.9 mm, 6.1 z-score) and normalization of RCA dimensions. Follow up cardiac CT (Day 130) demonstrated continued improvement in LAD aneurysm (2.6 mm). Trends in CRP and coronary artery z scores can be found in Fig. 3.

## Discussion and conclusion

We describe a case which illustrates that although KD is the most likely cause of CA aneurysm in children, there are other causes. Our patient was initially diagnosed with incomplete KD and progressive CA aneurysm enlargement over the first 6 weeks of illness. Her clinical course was atypical for KD, in that she had recurrent fever and inflammation over > 2 months with inability to wean from steroids. Even in the absence of treatment, fever is self-limited in KD and rarely lasts beyond 3–4 weeks [1]. The unusual nature of her course eventually led to consideration of a broader differential diagnosis, and additional imaging resulted in the finding of large vessel vasculitis. In KD patients with large/giant CA aneurysms, other medium-sized systemic arteries can develop discrete aneurysms, including the axillary, subclavian, brachial, femoral, iliac, splanchnic, and mesenteric arteries, usually near or at branching points. Diffuse aortitis/large artery inflammation without aneurysm is not seen in KD and this was a primary factor leading to consideration of alternative diagnoses, particularly TA [1, 4, 5]. With the diagnosis of TA, the anti-inflammatory treatment regimen was altered which led to clinical improvement and the CA aneurysms stabilizing and eventually regressing. Although the vast majority of CA aneurysms in children are related to KD, there are other rare etiologies. Etiologies other than KD should be considered in children whose clinical course is not consistent with KD, as in our patient, to expedite diagnosis and lessen progression in CA aneurysm.

Takayasu arteritis is an idiopathic large vessel vasculitis that primarily affects the aorta and its main branches [6]. Though it is more common in young women in the second and third decades of life, TA can also occur in children and infants [7]. The etiology is unknown, but it is thought that granulomatous inflammation and endothelial damage of the media and adventitia lead to fibrosis, scarring, stenosis, and more rarely may also cause aneurysm formation [8]. Symptoms of large vessel vasculitis can be difficult to detect until progressive stenosis leads to claudication, unless there are prominent constitutional symptoms. Furthermore, a lack of sensitive biomarkers makes the disease challenging to diagnose. Childhood TA is currently diagnosed with a combination of clinical findings, including pulse deficit or claudication, blood pressure discrepancies, bruits, and/or systemic hypertension, elevated acute phase reactants, and angiographic abnormalities of the aorta or its main branches, with MRA being the preferred imaging modality [4].

Coronary artery involvement, particularly CA stenosis, is fairly common in TA. Yet TA is rarely considered in infants and young children with CA aneurysm due to the low incidence of TA in this age range. The prevalence of CA involvement ranges from 3 to 30% in prior reports [9–11]. Coronary artery stenosis, particularly of the ostia of the left main CA and proximal segments of the LAD and RCA, is the most common CA finding in TA [12]. However, the majority of the literature focuses on the adult population, and important differences may exist between adult and pediatric populations. In one recent retrospective cohort study of 1096 TA patients comparing adult and pediatric patients, CA aneurysm or dilation were considerably more prevalent in the pediatric population (55.6% vs 3.4%) [11]. This may be due to intrinsic differences in vascular biology or differences in the inflammatory processes in children compared to adults. Delayed diagnosis of TA in children is common, with studies showing that the lag time between symptom onset and diagnosis is several-fold longer in children than adults [13]. Takayasu’s arteritis is associated with significant morbidity and mortality in both children and adults, with poorer prognosis in those with CA complications [9–13].

In addition to KD and TA, there are several other reported etiologies of CA dilation/aneurysm in children. As these diseases are rare causes of CA aneurysm, the literature mainly consists of case series and reports. Multiple autoimmune diseases have been implicated as potential causes of CA dilation/aneurysm. Polyarteritis nodosa has been associated with CA aneurysms in several case reports, with acute myocardial infarction as the presenting symptom of in several reports [14–18]. In a small case series of children with systemic juvenile idiopathic arthritis, coronary artery dilation (z-score > 2) was observed for 5 of the 12 patients at the time of presentation with fever [19]. All patients had CA dilation or small CAA (z-score < 5) and CA changes were transient (resolved within 4 months in all cases). Coronary artery dilation has been described as a rare finding at presentation in children with systemic lupus erythematosus and appears to improve with effective treatment [20, 21]. Coronary dilation or aneurysm have also been reported in young adults with granulomatosis with polyangiitis, microscopic polyangiitis and giant cell arteritis [22, 23]. Infectious illnesses, specifically Epstein Barr virus and Rickettsia infection [24, 25], as well as genetic conditions such as Noonan syndrome, connective tissue disorders, and neurofibromatosis type 1 [26–29], are rare causes of CA dilation. Lastly, there is evidence that febrile illnesses regardless of the underlying cause may lead to mild increases in CA dimensions. A study of children with acute, non-KD febrile illnesses showed CA dimensions were larger than afebrile children and a small number met definition of CA dilation (CA z-score 2–2.5 in 2/43, 5%) [30]. The pathogenesis of CA dilation in non-vasculitic febrile illness remains unknown, with hypothesized mechanisms including higher myocardial oxygen demand and resultant increase in coronary blood flow, and/or involvement of dysfunctional immune response pathways leading to an inflammatory milieu and high cytokine production [19, 31, 32].

Interest in etiologies of CA enlargement in children increased recently with the description of Multisystem Inflammatory Syndrome in Children (MIS-C), a newly characterized syndrome associated with SARS-CoV-2. In the case of our patient, multiple negative tests for SARs-CoV2 antibodies makes this diagnosis less likely. However, MIS-C has overlapping features with KD, including potential for CA dilation/aneurysm but also clear differences, including an older patient population, different ethnic distribution, more severe inflammation and myocardial involvement, and more frequent presentation in hypotensive shock [33–38]. Coronary artery changes have been described in MIS-C, with case series reporting an incidence of 5–20%. It is important to note that not all children with MIS-C and CA changes have stigmata of KD [36]. The majority of CA changes in MIS-C patients have been CA dilation or small CA aneurysms, but rare cases of giant aneurysms have also been described. The underlying pathology of CA changes in MIS-C has not been well described. Fever, inflammation and increased myocardial oxygen demand may play a role in cases of dilation but large or giant coronary aneurysms likely represent true pathologic changes to the arterial wall similar to KD and other vasculitis processes such as TA. Risk factors for CA changes in MIS-C have not yet been defined. Long-term prognosis and implications of CA changes in MIS-C have also not yet been described.

In summary, CA dilation or aneurysm, though most commonly associated with KD in children, can also be a manifestation of several other rheumatologic and infectious diseases. This case demonstrates the utility in broadening the differential diagnosis in cases of presumed KD with CA aneurysm but with a clinical course that is inconsistent with KD.

## Data Availability

Not applicable.

## Abbreviations

KD: Kawasaki disease
CA: Coronary artery
LAD: Left anterior descending coronary artery
RCA: Right coronary artery
TA: Takayasu arteritis
MIS-C: Multisystem Inflammatory Syndrome in Children
